# Dual orexin receptor antagonists for treatment of insomnia: A systematic review and meta-analysis on randomized, double-blind, placebo-controlled trials of suvorexant and lemborexant

**DOI:** 10.3389/fpsyt.2022.1070522

**Published:** 2022-12-12

**Authors:** Habibolah Khazaie, Masoud Sadeghi, Sepideh Khazaie, Max Hirshkowitz, Amir Sharafkhaneh

**Affiliations:** ^1^Sleep Disorders Research Center, Kermanshah University of Medical Sciences, Kermanshah, Iran; ^2^Medical Biology Research Center, Kermanshah University of Medical Sciences, Kermanshah, Iran; ^3^Students Research Committee, Kermanshah University of Medical Sciences, Kermanshah, Iran; ^4^Public Health Division, Department of Psychiatry, Stanford University School of Medicine, Palo Alto, CA, United States; ^5^Sleep Disorders and Research Center, Baylor College of Medicine, Houston, TX, United States

**Keywords:** insomnia, orexin receptor antagonist, suvorexant, lemborexant, randomized trial, meta-analysis

## Abstract

**Study objectives:**

Recent treatment guidelines for chronic insomnia recommend pharmacological and non-pharmacological therapies. One of the contemporary drug options for insomnia includes dual orexin receptor antagonist (DORA), such as suvorexant and lemborexant. We conducted a systematic review and meta-analysis for the treatment of insomnia with suvorexant and lemborexant based on randomized, double-blind, placebo-controlled Trials.

**Methods:**

We conducted a comprehensive search on three databases (PubMed/Medline, Web of Science, and Cochrane Library) till August 14, 2021, without any restrictions to retrieve the relevant articles. The effect sizes were computed presenting the pooled mean difference or risk ratio along with 95% confidence interval of each outcome.

**Results:**

Our search showed eight articles (five for suvorexant and three for lemborexant). Results of diary measures, rating scales, polysomnography results, treatment discontinuation, and adverse events were measured. All efficacy outcome measures favorably and significantly differed in the suvorexant compared to placebo. Safety profile did not differ significantly except for somnolence, excessive daytime sleepiness/sedation, fatigue, back pain, dry mouth, and abnormal dreams. Important adverse events including hallucinations, suicidal ideation/behavior and motor vehicle accidents did not differ between suvorexant and placebo. All the efficacy outcomes significantly differed between lemborexant 5 and lemborexant 10 compared to placebo. Somnolence rate for lemborexant 5 and lemborexant 10 and nightmare for lemborexant 10 were significantly higher than placebo.

**Conclusion:**

The present meta-analysis reported that suvorexant and lemborexant are efficacious and safe agents for the patients with insomnia. Further data in patients with insomnia and various comorbid conditions are needed.

## Introduction

Insomnia is a condition in which a person has difficulty falling asleep or staying asleep (1) with associated consequences like daytime fatigue and sleepiness (2–4). Insomnia is a major health challenge in the general population (5). Various studies around the world have reported that the insomnia was a high prevalent disease, affecting around 10–30% of the general population (1, 6, 7). Around 30–40% of adults in the US report insomnia symptoms at some point in a given year (8) and in China the prevalence of any type of insomnia symptoms was 22.1% (9). It is more common in the elderly, women, and people with medical and mental illness (6, 7, 10). There are three necessary diagnostic criteria for insomnia in clinical practice: complaint of trouble falling or staying asleep, adequate opportunity for sleep, and daytime dysfunction (11–14).

Recent treatment guidelines for chronic insomnia recommend pharmacological and non-pharmacological therapies (15). Current armamentarium of contemporary drug options for insomnia include gamma-aminobutyric acid type-A receptor agonists, non-benzodiazepine Z-drugs, sedative-hypnotic benzodiazepines, sedative antidepressants, melatonin receptor agonists, sedative antihistamines, and dual orexin receptor antagonists, such as suvorexant and lemborexant (15). The orexin/hypocretin system has been developed as a target for the treatment of insomnia (16). Orexins are neuropeptides that have a role in regulating the sleep-wake cycle by maintaining wakefulness (17).

Suvorexant is a dual orexin receptor antagonist that promotes sleep through selective antagonism of the endogenous oroxin-stimulating neuropeptides in the orexin receptors OX1R and OX2R (18, 19). Lemborexant is another dual orexin receptor antagonist and acts as a competitive antagonist in both orexin receptors, which can possibly stop the awakening by blocking binding of orexin (20).

Several meta-analyses reported the findings of clinical trials for the effectiveness of both suvorexant and lemborexant (21), suvorexant alone (22–26), and lemborexant alone (27, 28) in insomnia patients with different study population and results. We performed a meta-analysis among largest number of randomized, double-blind, placebo-controlled trials of suvorexant and lemborexant in insomnia patients to explore the efficacy and safety profile in more detail as it may relate to use of these agents in mental health clinics.

## Materials and methods

### Study design

The reporting of the present meta-analysis is in accordance with the preferred reporting items for systematic reviews and meta-analyses (PRISMA) protocols (29). The PICO (population, intervention, control, and outcomes) question was: Do suvorexant and lemborexant improve various relevant clinical efficacy and safety outcomes at various time points in patients with insomnia compared to placebo group? The outcomes of interest include diary measures, rating scales and adverse events.

### Identification of articles

A comprehensive search was performed by in three databases of PubMed/Medline, Web of Science, and Cochrane Library until August 14, 2021, without any restrictions to retrieve the relevant articles. The search strategy was (“suvorexant” or “lemborexant”) and (“insomnia”) and (“random*” or “trial*”). Moreover, the citations of the retrieved articles linked to the subject were examined to ensure that no study was missed and then the titles and abstracts of the relevant articles were evaluated; subsequently, the full texts of the articles following the eligibility criteria were downloaded.

### Inclusion and exclusion criteria

The inclusion criteria were: (1) published randomized, double-blind, placebo-controlled trials; (2) studies reporting outcomes of suvorexant or lemborexant compared to placebo in insomnia patients; (3) participants had age ≥ 18 years at the time of enrollment. We rejected the following study types: Meta-analyses, studies with incomplete data, studies without a placebo group, conference papers, reviews, case-reports, studies reporting the outcomes in healthy individuals, unpublished studies, book chapters, studies with duplicate data and comment.

### Data abstraction

One author (MS) extracted the data of the articles included in the meta-analysis. The extracted data were: first author, publication year, phase of the trial, duration of treatment, participants age range, type of treatment, sample size based on each outcome, and mean difference (MD) or risk ratio (RR) of each outcome. Another author (HK) re-checked them. Disagreements between the two authors were resolved by third author (AS).

### Statistical analysis

The effect sizes were computed using the Review Manager 5.3 (RevMan 5.3) presenting the MD or RR along with 95% confidence interval (CI) for each outcome. To estimate the pooled MD or RR significance, the Z-test was applied with a *p*-value (two-sided) less than 0.05 considered as significant. An I^2^ statistic (P_heterogeneity_ < 0.1 or I^2^ > 50%) showed a significant heterogeneity and random-effects model was performed (30), and if the heterogeneity was insignificant, a fixed-effect model was applied (31).

## Results

### Study selection

**FIGURE 1:**
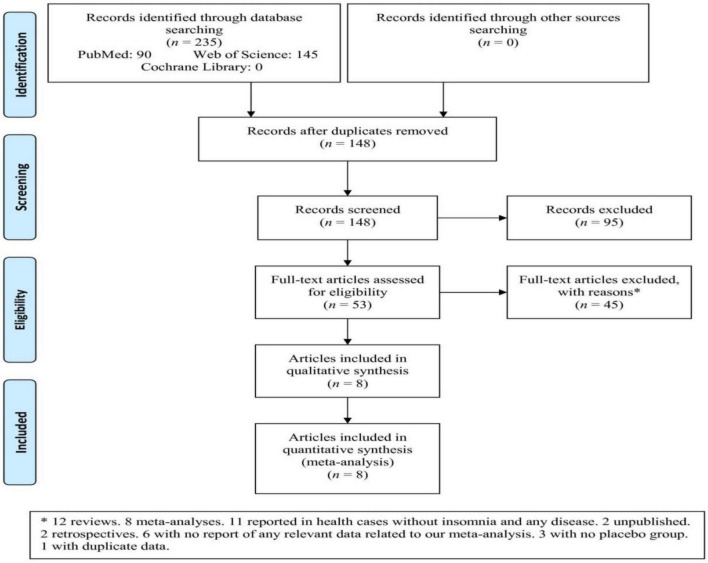
Flowchart of the study selection. *Twelve reviews. Eight meta-analyses. Eleven reported in health cases without insomnia and any disease. Two unpublished. Two retrospectives. Six with no report of any relevant data related to our meta-analysis. Three with no placebo group. One with duplicate data.

Among three databases, 235 articles were retrieved that after removing duplicates and irrelevant articles based on titles/abstracts, 53 full-text articles were assessed (Figure 1). Subsequently, 45 articles were excluded based on the reasons (12 reviews, eight meta-analyses, 11 reported in health cases without insomnia and any disease, two unpublished, two retrospectives, six with no report of any relevant data related to our meta-analysis, three with no placebo group, and one with duplicate data). Finally, eight articles were included in the meta-analysis that one article involved four independent studies (23, 32–38).

### Characteristics of the studies

The meta-analysis included 11 studies based on eight articles (Table 1). All articles were registered in Clinical trials.gov, except for one (32). Six articles were phase 3 (32, 33, 35, 36, 38, 39), and two were phase 2 trials (34, 37). All participants in the studies were age 18 years or older. Five articles including eight studies reported suvorexant therapy (32–34, 36, 39). Three publications with three studies reported lemborexant therapy in insomnia patients (35, 37, 38).

**TABLE 1:** Characteristics of articles included in the meta-analysis.

References	https://clinicaltrials.gov/ identifier	Phase	Duration	Age range, years	Reported treatment
Herring et al. (34)	NCT00792298	2	1 month	18–64	Suvorexant
Michelson et al. (36)	NCT01021813	3	1 year	≥18	Suvorexant
Herring et al. (23)*	NCT01097616 and NCT01097629	3	3 months	<65 and ≥65	Suvorexant
Fan et al. (32)	–	3	6 months	18–64	Suvorexant
Murphy et al. (37)	NCT01995838	2	6 months	19–80	Lemborexant
Rosenberg et al. (38)	NCT02783729	3	1 month	55–88	Lemborexant
Herring et al. (33)	NCT02750306	3	1 month	50–90	Suvorexant
Kärppä et al. (35)	NCT02952820	3	6 months	≥18	Lemborexant

*This article included four independent studies.

### Efficacy measures for suvorexant vs. placebo

#### Results of diary measures for suvorexant therapy

**FIGURE 2:**
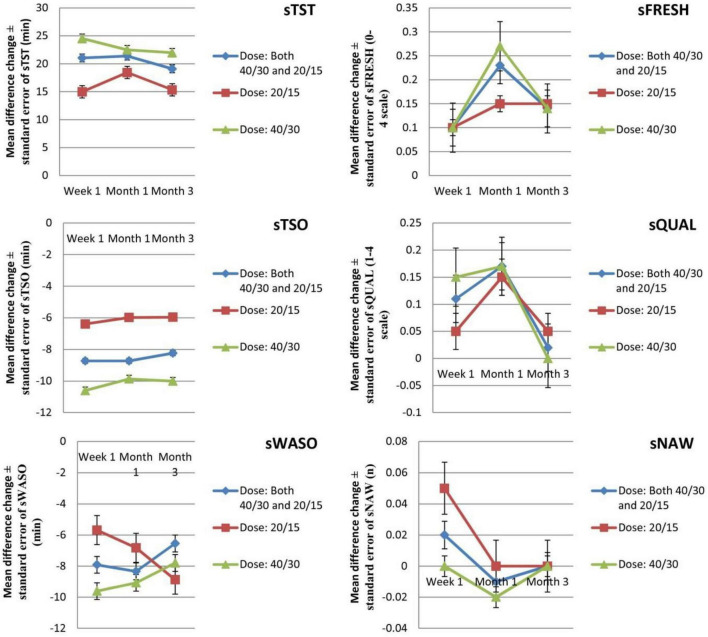
The mean changes efficacy outcome (diary measures) results from baseline in suvorexant vs. placebo for three times. sFRESH, subjective refreshed feeling on waking (0–4 scale); sNAW, subjective number of awakenings; sQUAL, subjective quality of sleep (1–4 scale); sTSO, subjective time to sleep onset (minutes); sTST, subjective total sleep time (minutes); sWASO, subjective wake after sleep onset (minutes); WMD, weighted mean difference.

Six efficacy outcomes [subjective total sleep time (sTST), subjective time to sleep onset (sTSO), subjective wake after sleep onset (sWASO), subjective quality of sleep (sQUAL), subjective number of awakenings (sNAW), and subjective refreshed feeling on waking (sFRESH)] were measured in the individuals (Tables 2, 3) during week 1 and months 1 and 3. All outcomes significantly differed in the suvorexant compared with placebo, with the exception of sQUAL at month 3, sNAW at week 1, sNAW at month 1, and sNAW at month 3. Figure 2 shows efficacy outcome results for suvorexant vs. placebo for three times.

**TABLE 2:** The mean changes of efficacy outcome (diary measures) results from baseline in suvorexant vs. placebo.

Diary measures	*N*	No. of cases (suvorexant/placebo)	WMD	95% CI	*P*-value	I^2^, %
sTST at week 1	5	1,739/1,732	21.05	16.00, 26.10	<0.00001	64
sTST at month 1	6	1,719/1,724	21.39	18.17, 24.61	<0.00001	0
sTST at month 3	4	1,113/1,328	19.10	14.63, 23.56	<0.00001	46
sTSO at week 1	5	1,739/1,732	–8.72	−11.04, −6.39	<0.00001	40
sTSO at month 1	6	1,719/1,724	–8.72	−11.03, −6.41	<0.00001	0
sTSO at month 3	4	1,113/1,328	–8.23	−10.92, −5.55	<0.00001	25
sWASO at week 1	4	1,231/1,480	–7.91	−10.21, −5.60	<0.00001	18
sWASO at month 1	5	1,683/1,675	–8.33	−10.70, −5.96	<0.00001	0
sWASO at month 3	4	1,113/1,328	–6.45	−9.20, −3.70	<0.00001	0
sQUAL at week 1	4	1,231/1,480	0.11	0.05, 0.17	0.0004	38
sQUAL at month 1	6	1,738/1,731	0.17	0.12, 0.22	<0.00001	0
sQUAL at month 3	4	1,113/1,328	0.02	−0.12, 0.17	0.74	81
sNAW at week 1	4	1,231/1,480	0.02	−0.40, 0.09	0.52	0
sNAW at month 1	5	1,683/1,675	–0.01	−0.07, 0.05	0.72	18
sNAW at month 3	4	1,113/1,328	0.00	−0.06, 0.06	1.00	0
sFRESH at week 1	4	1,231/1,480	0.10	0.06, 0.14	<0.00001	0
sFRESH at month 1	5	1,638/1,675	0.23	0.17, 0.29	<0.00001	34
sFRESH at month 3	4	1,113/1,328	0.14	0.08, 0.21	<0.0001	0

95% CI, 95% confidence interval; *N*, number of comparisons/studies; sFRESH, subjective refreshed feeling on waking (0–4 scale); sNAW, subjective number of awakenings; sQUAL, subjective quality of sleep (1–4 scale); sTSO, subjective time to sleep onset (minutes); sTST, subjective total sleep time (minutes); sWASO, subjective wake after sleep onset (minutes); WMD, weighted mean difference.

**TABLE 3:** Analysis of the mean changes of efficacy outcomes from rating scales and polysomnography from baseline in suvorexant vs. placebo.

	*N*	No. of cases (suvorexant/placebo)	WMD	95% CI	*P*-value	I^2^, %
**Rating scale**						
ISI at month 1	6	1,726/1,715	–1.50	−1.78, −1.22	<0.00001	26
ISI at month 3	4	1,106/1,318	–1.50	−1.88, −1.11	<0.00001	46
CGI-S at week 2	4	1,173/1,408	–0.38	−0.45, −0.30	<0.00001	0
CGI-S at month 1	6	1,812/1,802	–0.38	−0.44, −0.31	<0.00001	13
CGI-S at month 3	4	1,106/1,318	–0.38	−0.53, −0.22	<0.00001	76
PGI-S at week 2	4	1,173/1,408	–0.42	−0.49, −0.35	<0.00001	44
PGI-S at month 1	5	1,670/1,659	–0.40	−0.50, −0.30	<0.00001	65
PGI-S at month 3	4	1,106/1,318	–0.37	−0.46, −0.29	<0.00001	14
CGI-I at week 2	4	1,173/1,408	–0.40	−0.48, −0.32	<0.00001	22
CGI-I at month 1	5	1,670/1,659	–0.40	−0.47, −0.33	<0.00001	0
CGI-I at month 3	4	1,106/1,318	–0.45	−0.53, −0.37	<0.00001	0
PGI-I at week 2	4	1,173/1,408	–0.47	−0.58, −0.35	<0.00001	54
PGI-I at month 1	5	1,670/1,659	–0.48	−0.56, −0.41	<0.00001	0
PGI-I at month 3	4	1,106/1,318	–0.42	−0.54, −0.29	<0.00001	55
**Polysomnography**						
TST at month 1	2	199/193	29.47	14.13, 44.81	0.0002	0
LPS at day 1	5	950/1,275	–13.40	−18.20, −8.59	<0.00001	57
LPS at month 1	6	1,046/1,345	–11.07	−13.88, −8.26	<0.00001	0
LPS at month 3	4	815/1,012	–6.37	−9.44, −3.31	<0.0001	35
WASO at day 1	4	916/1,140	–37.63	−41.04, −34.22	<0.00001	23
WASO at month 1	5	1,014/1,227	–25.80	−29.48, −22.12	<0.00001	0
WASO at month 3	4	815/1,006	–24.97	−31.26, −18.68	<0.00001	60
**Response rate**						
ISI responders at month 3	4	1,067/1,276	1.31	1.20, 1.42	<0.00001	0

95% CI, 95% confidence interval; CGI-I, clinical global impression-improvement scale (1–7 scale); CGI-S, clinical global impression-severity scale (1–7 scale); ISI, insomnia severity index (0–28 scale); TST, total sleep time (minutes); LPS, latency to onset of persistent sleep (minutes); PGI-I, patient global impression-improvement scale (1–7 scale); PGI-S, patient global impression-severity scale (0–5 scale); *N*, number of comparisons/studies; RR, risk ratio; WASO, wakefulness after persistent sleep onset (minutes); WMD, weighted mean difference. The percentage of patients who had a clinically meaningful improvement (responders), prospectively defined as ≥6-point improvement from baseline in ISI score (40).

#### Rating scales and polysomnography results for suvorexant therapy

The studies compared six rating scales [insomnia severity index (ISI), clinical global impression-severity scale (CGI-S), patient global impression-severity scale (PGI-S), clinical global impression-improvement scale (CGI-I), and patient global impression-improvement scale (PGI-I)], three outcomes from polysomnography [TST, latency to onset of persistent sleep (LPS), and WASO], and ISI responders. For the rating scales, ISI, CGI-S, PGI-S, CGI-I, and PGI-I for several time points were calculated. For polysomnography, TST at month 1 and LPS and WASO at day 1 and months 1 and 3 were calculated. ISI responders [the percentage of patients who had a clinically meaningful improvement (responders), prospectively defined as ≥ 6-point improvement from baseline in ISI score] were measured in month 3 (40). All outcomes from rating scales and polysomnography differed significantly between the two study groups.

### Treatment discontinuation and adverse events for suvorexant therapy vs. placebo

The results of outcomes of treatment discontinuation and adverse events are shown in Table 4. The mean changes from baseline in weight and proportions of patients with ≥ 7% increase or decrease in weight (weight: ≥ 7%) has been calculated in two times intervals. Although discontinuation rates did not differ between the two study groups, several safety outcomes were significantly higher in suvorexant compared to placebo for at least one drug-related adverse event, somnolence, excessive daytime sleepiness/sedation, fatigue, abnormal dreams, back pain, and dry mouth.

**TABLE 4:** Treatment discontinuation and individual adverse events for suvorexant vs. placebo.

Adverse events	*N*	No. of cases (suvorexant/placebo)	RR	95% CI	*P*-value	I^2^, %
Discontinuation due to all cause*	7	1,978/1,218	0.95	0.82, 1.10	0.48	0
Discontinuation due to intolerability*	7	1,978/1,218	1.00	0.74, 1.35	1.00	29
Discontinuation due to inefficacy*	7	1,978/1,218	0.75	0.52, 1.09	0.13	0
≥1 adverse event	7	1,986/1,228	1.07	0.99, 1.15	0.07	0
≥1 drug-related adverse event**	7	1,986/1,228	1.62	1.40, 1.89	<0.00001	10
≥1 serious adverse event	7	1,986/1,228	0.70	0.27, 1.84	0.47	52
Discontinued owing to adverse event	3	723/461	1.37	0.88, 2.13	0.16	0
Somnolence	7	1,986/1,228	3.26	2.29, 4.63	<0.00001	0
Excessive daytime sleepiness/sedation***	5	1,784/1,025	3.48	1.13, 10.67	0.03	0
Fatigue	6	1,844/1,085	2.09	1.28, 3.43	0.003	3
Cataplexy	6	1,926/1,168	Not estimable	–	−	–
Sleep paralysis	6	1,926/1,168	2.84	0.49, 16.35	0.24	0
Complex sleep–related behaviors	6	1,926/1,168	1.66	0.17, 15.84	0.66	0
Hypnagogic/hypnopompic hallucination	6	1,926/1,168	3.12	0.67, 14.47	0.15	0
Abnormal dreams	4	1,263/767	2.91	1.12, 7.60	0.03	0
Suicidal ideation/behavior	6	1,926/1,168	1.91	0.46, 7.92	0.38	20
Events indicative of abuse potential****	6	1,926/1,168	1.08	0.69, 1.68	0.74	0
Fall^#^	6	1,926/1,168	1.03	0.56, 1.89	0.92	19
Headache	7	1,986/1,228	1.13	0.86, 1.47	0.39	0
Dizziness	6	1,844/1,685	1.62	0.57, 4.57	0.36	85
Back pain	5	1,784/1,025	0.51	0.27, 0.97	0.04	0
Dry mouth	7	1,986/1,228	2.15	1.23, 3.76	0.008	0
Nasopharyngitis	5	1,784/1,025	0.95	0.71, 1.28	0.75	0
Motor vehicle accidents/violations^##^	5	1,660/963	1.21	0.72, 2.05	0.47	11
Sleep-onset paralysis	2	663/401	1.49	0.06, 36.41	0.81	–
Weight: ≥7% increase at month 3	4	1,250/1,514	1.38	0.78, 2.45	0.26	0
Weight: ≥7% decrease at month 3	4	1,250/1,514	1.85	0.84, 4.11	0.13	0

95% CI, 95% confidence interval; *N*, number of comparisons/studies; RR, risk ratio; weight: ≥7%, the mean changes from baseline in weight and proportions of patients with ≥ 7% increase or decrease in weight.

*The counts for discontinuations due to adverse events are based on the period in which the adverse event started.

**Drug-related adverse event a determined by the investigator to be related to the drug (determination made while blinded).

***Excessive daytime sleepiness was defined as a more persistent daytime sleepiness than typical next-day residual somnolence.

****Terms included depersonalization, derealization, dissociation, euphoric mood, mania, hallucination, and potential study medication misuse.

^#^Falls were adjudicated to determine whether they were suggestive of cataplexy.

^##^Includes spontaneously reported events when the patient was the driver and events elicited *via* a motor vehicle accidents and violations questionnaire.

### Efficacy measures for lemborexant vs. placebo

#### Diary measures results for lemborexant 5 therapy

Three outcomes [subjective sleep onset latency (sSOL), subjective sleep efficiency (sSE), and sWASO] were measured in the individuals with insomnia (Table 5) during first seven nights and month 1. All the outcomes significantly differed between the lemborexant 5 compared to placebo.

**TABLE 5:** The mean changes of efficacy outcome (diary measures) results from baseline in lemborexant 5 vs. placebo.

Diary measures	*N*	No. of cases (lemborexant 5/placebo)	WMD	95% CI	*P*-value	I^2^, %
sSOL at first seven nights	2	569/516	–12.05	−18.45, −5.55	0.0003	79
sSOL at month 1	2	550/496	–11.80	−21.70, −1.90	0.02	88
sSE at first seven nights	2	624/511	4.10	2.88, 5.32	<0.00001	0
sSE at month 1	2	543/490	3.16	0.84, 5.49	0.008	52
sWASO at first seven nights	2	571/516	–13.19	−19.33, −7.26	<0.0001	0
sWASO at month 1	2	551/596	–6.74	−13.12, −0.37	0.04	0

95% CI, 95% confidence interval; *N*, number of comparisons/studies; sSOL, subjective sleep onset latency (minutes); sSE, subjective sleep efficiency (percentage); sWASO, subjective wake after sleep onset (minutes); WMD, weighted mean difference.

#### Results of diary measures for lemborexant 10 therapy

The outcomes of sSOL, sSE, and sWASO were measured in the insomnia patients (Table 6) during first seven nights and month 1. All the outcomes showed significant difference between the lemborexant 10 compared to placebo.

**TABLE 6:** The mean changes of efficacy outcome (diary measures) results from baseline in lemborexant 10 vs. placebo.

Diary measures	*N*	No. of cases (lemborexant 10/placebo)	WMD	95% CI	*P*-value	I^2^, %
sSOL at first seven nights	2	576/516	–12.45	−16.94, −7.96	<0.00001	60
sSOL at month 1	2	555/496	–13.11	−19.37, −6.85	<0.0001	71
sSE at first seven nights	2	564/511	6.32	4.99, 7.64	<0.00001	3
sSE at month 1	2	541/490	5.54	1.52, 9.56	0.007	81
sWASO at first seven nights	2	572/516	–21.38	−31.51, −11.25	<0.0001	62
sWASO at month 1	2	550/496	–13.77	−28.65, 1.11	0.07	75

95% CI, 95% confidence interval; *N*, number of comparisons/studies; sSOL, subjective sleep onset latency (minutes); sSE, subjective sleep efficiency (percentage); sWASO, subjective wake after sleep onset (minutes); WMD, weighted mean difference.

Supplementary Table 1 shows the outcomes of sTST, sSE, and sWASO in the insomnia patients during first seven nights and month 1 comparing 5 and 10 mg doses of lemborexant. None of the outcomes differed significantly between the comparison groups, with the exceptions of sSE at First seven nights and sSE at month 1.

### Treatment discontinuation and adverse events for leborexant therapy

Table 7 shows treatment discontinuation and individual adverse events for lemborexant 5 compared with placebo. There were only significant differences between two groups (lemborexant 5 and placebo) for any treatment-related treatment-emergent adverse event (TEAE) and somnolence.

**TABLE 7:** Treatment discontinuation and individual adverse events for lemborexant 5 vs. placebo.

Adverse events	*N*	No. of cases (lemborexant 5/placebo)	RR	95% CI	*P*-value	I^2^, %
Discontinuation due to all cause	2	589/534	1.02	0.76, 1.37	0.91	20
Discontinuation due to intolerability	2	589/534	1.06	0.45, 2.46	0.90	0
Any TEAE	3	618/584	1.01	0.90, 1.13	0.86	0
Any treatment-related TEAE	3	618/584	1.69	1.29, 2.22	0.0001	0
Any severe TEAE	2	580/528	1.05	0.50, 2.20	0.89	43
Any serious TEAE	3	618/584	1.46	0.55, 3.90	0.45	0
Any TEAE leading to discontinuation of study drug	3	618/584	1.05	0.51, 2.15	0.89	0
Any TEAE leading to interruption of study drug	2	580/528	1.73	0.74, 4.07	0.21	0
Death	2	580/528	Not estimable	–	−	–
Somnolence	3	618/584	4.05	2.14, 8.06	<0.0001	0
Headache	3	618/584	1.24	0.82, 1.87	0.31	0
Upper respiratory tract infection	2	580/528	1.28	0.65, 2.52	0.48	0
Back pain	2	352/375	0.68	0.28, 1.63	0.39	–
Urinary tract infection	2	580/528	0.73	0.27, 1.96	0.53	0
Nightmare	2	352/375	4.16	0.69, 25.20	0.12	0
Abnormal dreams	2	352/375	1.39	0.51, 3.78	0.52	0
Dizziness	2	304/365	0.59	0.13, 2.60	0.49	–

95% CI, 95% confidence interval; *N*, number of comparisons/studies; RR, risk ratio; TEAE, treatment-emergent adverse event. A TEAE was defined as an adverse event with onset date on or after the first dose of study drug up to 14 days after the last dose of study drug. Participants with two or more TEAEs with the same preferred term are counted only once for that preferred term.

Table 8 shows treatment discontinuation and individual adverse events for lemborexant 10 compared with placebo. There were only significant differences between two groups (lemborexant 10 and placebo) for any treatment-related TEAE, somnolence, and nightmare.

**TABLE 8:** Treatment discontinuation and individual adverse events for lemborexant 10 vs. placebo.

Adverse events	*N*	No. of cases (lemborexant 10/placebo)	RR	95% CI	*P*-value	I^2^, %
Discontinuation due to all cause	2	591/534	1.30	0.98, 1.71	0.07	54
Discontinuation due to intolerability	2	591/534	1.83	0.86, 3.87	0.12	0
Any TEAE	3	614/584	1.15	0.87, 1.53	0.33	0.69
Any treatment-related TEAE	3	614/584	2.08	1.61, 2.70	<0.00001	0
Any severe TEAE	2	582/528	0.74	0.33, 1.66	0.46	0
Any serious TEAE	3	618/584	1.46	0.55, 3.90	0.45	0
Any TEAE leading to discontinuation of study drug	3	614/584	1.60	0.58, 4.39	0.36	0
Any TEAE leading to interruption of study drug	2	582/528	0.99	0.39, 2.50	0.97	0
Death	2	582/528	Not estimable	–	–	–
Somnolence	3	614/584	6.48	3.35, 12.56	<0.00001	0
Headache	3	614/584	0.97	0.62, 1.50	0.88	0
Upper respiratory tract infection	2	582/528	0.65	0.11, 3.98	0.64	60
Back pain	2	346/375	1.32	0.55, 3.19	0.53	0
Urinary tract infection	2	582/528	1.84	0.82, 4.13	0.14	14
Nightmare	2	346/375	8.46	1.56, 45.78	0.01	0
Abnormal dreams	2	346/375	2.12	0.12, 35.91	0.60	70
Dizziness	2	241/324	0.64	0.12, 3.47	0.61	–

95% CI, 95% confidence interval; *N*, number of comparisons/studies; RR, risk ratio; TEAE, treatment-emergent adverse event. A TEAE was defined as an adverse event with onset date on or after the first dose of study drug up to 14 days after the last dose of study drug. Participants with two or more TEAEs with the same preferred term are counted only once for that preferred term.

### Treatment discontinuation and adverse events for lemborexant 5 and 10 therapies

Supplementary Table 2 shows treatment discontinuation and individual adverse events for lemborexant 5 compared to lemborexant 10. There were only significant differences in somnolence and urinary tract infection between two groups (lemborexant 5 and lemborexant 10) for any TEAE leading to discontinuation of study drug.

## Discussion

The present study reports on meta-analysis of suvorexant or lemborexant compared with placebo for the treatment of insomnia patients. Overall, both medications were significantly more efficacious compared to placebo. Suvorexant was superior to placebo with regard to the primary efficacy outcomes (sTST, sTSO, sWASO, sQUAL, and sFRESH). Suvorexant was also superior to placebo regarding outcomes from rating scales and polysomnography, with the exceptions of weight at month 3. This study showed that there were significant differences between suvorexant and placebo in the most adverse events, with the exception of somnolence, excessive daytime sleepiness/sedation, fatigue, back pain, dry mouth, and abnormal dreams that their prevalence in suvorexant group was higher than placebo group. In addition, lemborexant 5 and 10 was superior to placebo with regard to the primary efficacy outcomes (sTST, sSE, and sWASO), with the exception of sWASO at month 1 for lemborexant 10, and also there was no significant difference between lemborexant 5 and 10 and placebo in the most adverse events, with the exception of somnolence for lemborexant 5 and 10 and nightmare for lemborexant 10 was higher than placebo group. Lemborexant 10 was superior to lemborexant 5 with regard to sSE that being less of somnolence and urinary tract infection in Lemborexant 10 confirms superiority of Lemborexant 10 compared to lemborexant 5.

In the evaluation of suvorexant, attention was paid to the side effects that could be based on the mechanism. Narcolepsy is associated with a decrease in orexin neurons and possibly an orexinergic tone (41–43), raising the theoretical likelihood that blocking the orexin receptors may mimic the signs or symptoms of narcolepsy, especially cataplexy (23). No events adjudicated as cataplexy were observed in the present meta-analysis. Surveillance has been associated with a small number of reports of sleep-related hallucinations and sleep paralysis, both of which can happen spontaneously in the general population (44, 45). In the present meta-analysis, there was no significant difference between suvorexant and placebo regarding hypnagogic/hypnopompic hallucination, sleep paralysis, and sleep-onset paralysis. Although orexin has been hypothesized to regulate weight (46), no difference with placebo in weight change was observed in the present meta-analysis.

The US Food and Drug Administration (FDA) has stated that their overall approach to insomnia medications is to use the lowest effective dose of suvorexant (47). Although one trial and the present meta-analysis based on the results of diary measures showed the 30 or 40 mg dose of suvorexant to be generally suitable and well-tolerated in insomnia patients, but the FDA concluded that the safety and tolerability data across the development programs, including results from driving studies in healthy participants, did not support the use of the 30 or 40 mg dose for the treatment of insomnia (36). Therefore, the FDA suggested that the totality of the clinical data supported the use of lower suvorexant doses of 10–20 mg (47). However, in the next trials, it is necessary to focus on the role of drug dosage in insomnia patients more carefully and better.

Studies have shown that treatment with lemborexant may alleviate some of the changes in sleep architecture seen in older people with insomnia (28, 35). In particular, the increase in TST and stage R sleep with lemborexant therapy illustrated in polysomnography recordings was consistent with the self-reported improvement in sleep retention associated with lemborexant in comparison with placebo. The present meta-analysis confirmed these results. Increased rates of somnolence showed evidence of dose-response in the lemborexant groups compared with placebo and increased rates of somnolence and urinary tract infection in lemborexant 10 compared with lemborexant 5 showed evidence of dose-response in the treatment of insomnia patients. However, sSE levels in lemborexant 10 was higher than lemborexant 5. Therefore, the researchers should consider the dose-response of lemborexant in the future trials more cautious.

Our analysis has a number of strengths: data from randomized, double-blind, placebo-controlled trials, and the number of patients evaluated were relatively large. In contrast, several limitations should be acknowledged. First, studies in this analysis included different drug dosing regimens. Second, some studies included few cases and may not have included statistical comparisons between the groups. Third, the number of studies included is limited, outcome measures and time points for heterogeneous analysis, and for some outcome measures, only a few studies have been reported.

## Conclusion

The study showed that suvorexant and lemborexant are two efficacious and safe treatments for insomnia. As many patients with insomnia have significant comorbid conditions, further data in patients with insomnia and comorbid conditions are needed.

## A brief summary

### Current knowledge

Insomnia is a prevalent sleep disorder with various consequences. Pharmacotherapy and cognitive behavioral therapy for insomnia are main management strategies available. Dual orexin receptor antagonists are a new line of pharmacotherapeutic agents for management of insomnia. As DORA is a new line of therapy, practitioners may not be familiar with efficacy and side effects of these medications.

### Study impact

Our meta-analysis indicated that DORAs are efficacious and safe. Better awareness of the efficacy and safety of this new mechanistic line of insomnia treatment may help practitioners to better manage their patients. As majority of clinical trials exclude patients with significant comorbid conditions, further studies needed to evaluate safety and efficacy in patients with insomnia and various comorbid conditions.

## Data availability statement

The original contributions presented in this study are included in the article/Supplementary material, further inquiries can be directed to the corresponding authors.

## Author contributions

HK: supervision of all steps, study design, and manuscript writing. MS: databases search, data analysis, and manuscript writing. SK: manuscript writing and editing and data analysis. MH: supervision of all steps, editing the manuscript, and scientific writing. AS: first idea, supervision, and manuscript writing and editing. All authors contributed to the article and approved the submitted version.
