# Supplementary material for: Dual orexin receptor antagonists for treatment of insomnia: A systematic review and meta-analysis on randomized, double-blind, placebo-controlled trials of suvorexant and lemborexant
Source: Front Psychiatry. 2022 Dec 12;13:1070522. doi: 10.3389/fpsyt.2022.1070522 (PMC9792135; doi:10.3389/fpsyt.2022.1070522)
Supplement: Supplementary file 1 [file Data_Sheet_1.PDF]

Supplemental Data

Dual Orexin Receptor Antagonists for Treatment of Insomnia: A Systematic Review and Meta-analysis on Randomized, Double-blind, Placebo-controlled Trials of Suvorexant and Lemborexant

Habibolah Khazaie, MD (hakhazaie@kums.ac.ir)[1], Masoud Sadeghi, PhD (Sadeghi_mbrc@yahoo.com)[2&3], Sepideh Khazaie, MD (Sepidehkhazaieee@gmail.com)[1], Max Hirshkowitz, PhD (maxh@gmail.com)[4&5], Amir Sharafkhaneh, MD, PhD (amirs@bcm.edu)[5]

[1] Sleep Disorders and Research Center, Kermanshah University of Medical Sciences, Kermanshah, Iran

[2] Medical Biology Research Center, Kermanshah University of Medical Sciences, Kermanshah, Iran

[3] Students Research Committee, Kermanshah University of Medical Sciences, Kermanshah, Iran

[4] Stanford University School of Medicine Department of Psychiatry, Public Health Division, Palo Alto, California.

[5] Sleep Disorders and Research Center, Baylor College of Medicine, Houston, TX USA

Corresponding author

Amir Sharafkhaneh, MD, PhD

Professor of Medicine, Baylor College of Medicine

Senior Physician, Sleep Disorders and Research Center

Michael E. DeBakey VA Medical Center

Houston, TX

Table 1S: The mean changes of efficacy outcome (diary measures) results from baseline in lemborexant 5 versus lemborexant 10

Diary Measures	N	No. of cases (Lemborexant 5/ Lemborexant 10)	WMD	95%CI	P-value	I^2, %
sSOL at First 7 nights	2	569/576	0.84	-2.03, 3.71	0.57	0
sSOL at Month 1	2	550/555	2.16	-1.14, 5.46	0.20	1
sSE at First 7 nights	2	624/564	-2.16	-3.37, -0.95	0.0005	42
sSE at Month 1	2	543/541	-2.11	-3.73, -0.49	0.01	0
sWASO at First 7 nights	2	571/572	8.54	-4.94, 22.01	0.21	75
sWASO at Month 1	2	551/550	6.55	-5.66, 18.75	0.29	67

Abbreviations: 95% CI, 95% confidence interval; N, number of comparisons/studies; sSOL, subjective sleep onset latency (minutes); sSE, subjective sleep efficiency (percentage); sWASO, subjective wake after sleep onset (minutes); WMD, weighted mean difference.

Table S2: Treatment discontinuation and individual adverse events for lemborexant 5 versus lemborexant 10

Adverse events	N	No. of cases (Lemborexant 5/ Lemborexant 10)	RR	95%CI	P-value	I^2, %
Discontinuation due to all cause	2	589/591	0.79	0.60, 1.03	0.08	0
Discontinuation due to intolerability	2	589/591	0.58	0.28, 1.20	0.14	0
Any TEAE	3	618/614	0.97	0.87, 1.09	0.61	26
Any treatment-related TEAE	3	618/614	0.81	0.66, 1.01	0.06	0
Any severe TEAE	2	580/582	1.40	0.63, 3.12	0.41	0
Any serious TEAE	3	618/614	1.00	0.41, 2.44	1.00	26
Any TEAE leading to discontinuation of study drug	3	618/614	0.52	0.28, 0.95	0.03	0
Any TEAE leading to interruption of study drug	2	580/582	1.71	0.74, 3.93	0.21	0
Death	2	580/582	Not estimable	-	-	-
Somnolence	3	618/614	0.62	0.43, 0.90	0.01	0
Headache	3	618/614	1.28	0.85, 1.94	0.23	0
Upper respiratory tract infection	2	580/582	1.90	0.89, 4.05	0.09	36
Back pain	2	352/346	0.80	0.33, 1.94	0.62	0
Urinary tract infection	2	580/582	0.39	0.16, 0.93	0.03	0
Nightmare	2	352/346	0.48	0.17, 1.38	0.17	0
Abnormal dreams	2	352/346	0.89	0.16, 5.05	0.90	51
Dizziness	2	304/241	1.18	0.20, 6.99	0.86	-

Abbreviations: 95% CI, 95% confidence interval; N, number of comparisons/studies; RR, risk ratio; TEAE, treatment-emergent adverse event. Note: A TEAE was defined as an adverse event with onset

date on or after the first dose of study drug up to 14 days after the last dose of study drug. Participants with two or more TEAEs with the same preferred term are counted only once for that preferred term.
